# Formulation-Property Effects in Novel Injectable and Resilient Natural Polymer-Based Hydrogels for Soft Tissue Regeneration

**DOI:** 10.3390/polym16202879

**Published:** 2024-10-12

**Authors:** Daniella Goder Orbach, Ilana Roitman, Geffen Coster Kimhi, Meital Zilberman

**Affiliations:** Department of Biomedical Engineering, Tel-Aviv University, Tel Aviv 6997801, Israel; goderdan@mail.tau.ac.il (D.G.O.); ilanaroitman@mail.tau.ac.il (I.R.); geffencoster@mail.tau.ac.il (G.C.K.)

**Keywords:** gelatin, resilience, porous hydrogel, tissue regeneration

## Abstract

The development of injectable hydrogels for soft tissue regeneration has gained significant attention due to their minimally invasive application and ability to conform precisely to the shape of irregular tissue cavities. This study presents a novel injectable porous scaffold based on natural polymers that undergoes in situ crosslinking, forming a highly resilient hydrogel with tailorable mechanical and physical properties to meet the specific demands of soft tissue repair. By adjusting the formulation, we achieved a range of stiffness values that closely mimic the mechanical characteristics of native tissues while maintaining very high resilience (>90%). The effects of gelatin, alginate, and crosslinker concentrations, as well as porosity, on the hydrogel’s properties were elucidated. The main results indicated a compression modulus range of 2.7–89 kPa, which fits all soft tissues, and gelation times ranging from 5 to 30 s, which enable the scaffold to be successfully used in various operations. An increase in gelatin and crosslinker concentrations results in a higher modulus and lower gelation time, i.e., a stiffer hydrogel that is created in a shorter time. In vitro cell viability tests on human fibroblasts were performed and indicated high biocompatibility. Our findings demonstrate that these injectable hydrogel scaffolds offer a promising solution for enhancing soft tissue repair and regeneration, providing a customizable and resilient framework that is expected to support tissue integration and healing with minimal surgical intervention.

## 1. Introduction

In situ tissue regeneration has emerged at the forefront of organ dysfunction treatment. Tissue regeneration requires the recruitment of native stem cells and their differentiation into the target tissue. For cells to achieve successful differentiation, they require a set of conditions that allow for the correct biochemical and biophysical cues [1]. Research in biomaterial design has been focused on providing these conditions with an easy-to-use scaffold. One key area of interest is in-situ-forming injectable scaffolds. These scaffolds offer several advantages [2].

One of the primary benefits of injectable hydrogels is their ability to be delivered through minimally invasive techniques. This is particularly advantageous in clinical settings where reducing patient trauma and recovery time is crucial. The injectable nature allows for the precise placement of the hydrogel within the target tissue or cavity, minimizing the need for extensive surgical procedures. The avoidance of invasive incisions not only leads to quicker patient recovery times but also to a better esthetic outcome by minimizing scarring. However, the injectable options available today are not suitable enough. Hyaluronic acid is commonly used as an injectable filler but is resorbed quickly, and repeat procedures are therefore necessary [3]. Autologous fat injection has also been tried, but if the fat has no scaffold to adhere to, it undergoes up to 70% resorption [4,5]. Therefore, developing a suitable injectable tissue scaffold remains an unmet need. The addition of an injectable scaffold that allows cell adherence and proliferation may solve these issues by providing a suitable environment for cell growth.

Tissue regeneration strongly relies on the mechanical properties of the surrounding environment. Over the past couple of decades, it has been established that mechanostimulation is highly dependent on the stiffness of the native biological tissue [1,6,7]. Different tissues have different stiffnesses, from the softness of neural tissue (under 50 Pa) to the rigidity of bone (2–4 GPa) [8]. Therefore, developing an appropriate scaffold with matching stiffness is crucial for promoting cellular growth and tissue regeneration.

**Figure 1:**
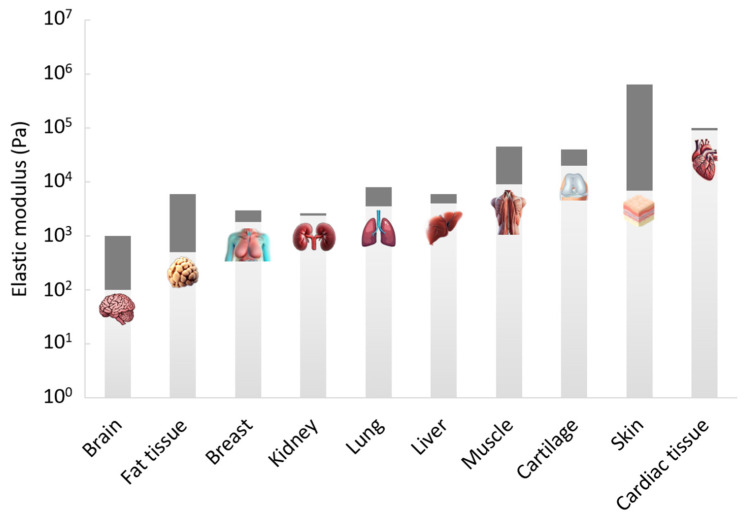
Elastic modulus of various soft tissues.

Soft tissues in the human body exhibit a wide range of elastic moduli, reflecting their diverse mechanical functions and structural roles (Figure 1). For instance, the elastic modulus of lung tissue is relatively low, ranging from 1 to 6 kPa, which supports its role in accommodating repetitive breathing motions and maintaining flexibility during respiration [9]. Adipose tissue, which serves as a cushion and energy store, also demonstrates low modulus values but varies depending on fat type and location [9]. For example, the modulus of subcutaneous fat is generally lower compared to visceral fat, reflecting different functional requirements and mechanical properties. On the other hand, tissues such as skin, which need to withstand external forces while maintaining elasticity, exhibit higher moduli, typically ranging from 10 to 100 kPa [9]. These variations in elastic modulus are crucial for the tissues’ ability to perform their specific biological functions effectively, from cushioning and flexibility to structural support and protection.

While scaffold stiffness is essential, it is not the only factor in effective scaffold design. Most tissues experience cyclic loads, either in compression or tension, and a scaffold must withstand these without significant plastic deformation. Several papers have reported synthetic hydrogel networks that are able to mimic the resilience of biological tissues; Wang et al. described porous polymer sponges based on 5-ethyl-5-(hydroxymethyl)-β, β-dimethyl-1, 3-dioxane-2-ethanol diallyl carbonate (EHDAC) and poly (ethylene glycol) diallyl carbonate (PEGDAC) that are fatigue resistant [10], and Han et al. reported a dual-crosslinked hydrogel adhesive based on acrylic acid and tri(ethylene glycol) diacrylate-dopamine that is highly resilient for wound healing applications [11]. Combinations of natural and synthetic polymers have also been investigated. A hydrogel based on cellulose in combination with poly(vinyl alcohol) was developed for cartilage tissue engineering [12], and a resilient adhesive for sealing gastric perforations was based on a combination of a gelatin-based network with an acrylamide-based hydrogel [13]. However, while synthetic polymers are relatively easy to control in terms of mechanical, physical, and chemical properties, they lack the bioactivity to support cell adhesion and proliferation [14].

Natural polymers have been widely used in biomedical applications due to their high biocompatibility, low immunogenicity, and relative ease of processing. Therefore, they present a better option for in vivo applications. Various scaffolds for tissue regeneration based on natural polymers have been reported. Marine collagen was found to be very diverse, and has been used to fabricate skin, cartilage, and bone tissue engineering scaffolds, but it has a denaturation temperature under 37 °C, making it not ideal for in vivo applications [15]. A hydrogel based on alginate, gelatin, and cellulose nanocrystals has been developed for bone tissue engineering and has shown good biocompatibility and bioactivity [16]. Chitosan-collagen-hydroxyapatite membranes for tissue engineering have been shown to have high biocompatibility and lead to good cellular adhesion [17], and hyaluronic acid/alginate and elastin/gelatin/hyaluronic acid inks were used to 3D print cartilage scaffolds [18,19]. However, these biomaterials either did not prove to have the resilience required for fatigue resistance or are too tough for soft tissue applications. Hence, developing injectable hydrogels based on natural polymers that possess the desired mechanical properties for soft tissue regeneration is considered to be a challenge.

We have developed a versatile platform of porous hydrogel structures that combines high biocompatibility and biodegradability with ease of customization to create injectable scaffolds for the regeneration of a wide range of soft tissues. These highly resilient porous scaffolds for soft tissue regeneration are based on the natural polymers’ gelatin and alginate and are reported in the current paper. Gelatin, a derivative of collagen, was chosen as the primary polymer because it facilitates cell adhesion and, when crosslinked, provides a hydrogel with the mechanical properties desired for soft tissue regeneration. Alginate, a seaweed-derived polysaccharide, is commonly used in the biomedical field. Although it does not possess the benefits of cell adhesion facilitation, it is also a biocompatible and biodegradable polymer. Both gelatin and alginate can be chemically crosslinked with N-(3-dimethylaminopropyl)-N-ethylcarbodiimide hydrochloride (EDC) to stabilize the hydrogel.

By changing the polymer concentrations, crosslinker concentration, and hydrogel porosity, we alter the mechanical and physical properties of the scaffolds. We focus on four basic formulations, varying in gelatin, alginate, and EDC concentrations, each one with various porosities, to elucidate the effect of each of the formulation parameters on the hydrogel’s characteristics. Such control over the hydrogel’s properties allows us to create a favorable environment for cellular infiltration and adhesion and present a scaffold for de novo tissue growth in vivo.

## 2. Materials and Methods

Hydrogels were prepared from cold-water fish skin gelatin (G7041, Sigma-Aldrich, Rehovot, Israel), alginic acid sodium salt (A1112, Sigma-Aldrich, Rehovot, Israel), and N-(3-dimethylaminopropyl)-N’-ethylcarbodiimide hydrochloride (EDC) (E7750, Sigma-Aldrich, Rehovot, Israel).

### 2.1. Hydrogel Preparation

Hydrogels were prepared by mixing a polymer solution with a crosslinking solution. The polymer solution was prepared by dissolving gelatin and alginate in double distilled water (DDW) at 60 °C for approximately 10 min, until a homogeneous solution was obtained, and then the solution was cooled to room temperature. A crosslinking solution was prepared by mixing various EDC concentrations in DDW until a homogeneous solution was obtained. The two solutions were loaded into a commercial double syringe fitted with a static mixer tip (Mixpac™, L-system 2.5 mL, 4:1 volume ratio, purchased from Sulzer Mixpac, AG, Haag, Switzerland), which homogeneously mixes the two solutions. As the two solutions are mixed by the static mixer, they begin to undergo chemical crosslinking, and the duration of crosslinking depends on the polymer and crosslinker concentrations. To create porous hydrogels, prior to loading the double syringe, the polymeric solution was foamed in the following manner: the polymer solution was loaded into a regular syringe, and a second syringe was loaded with air. Then, using a three-way stopcock, the polymer solution and air were mixed vigorously until the air was fully mixed with the polymer solution. The resulting polymer-air mixture was then loaded into the double syringe, and the process continued as described above. Hydrogels were tested at room temperature since cold-water fish gelatin is liquid at room temperature, and this would be close to clinical use conditions.

The studied hydrogel formulations are described in Table 1. Throughout this paper, they are presented in the form of Gel-Al-EDC (p:a), where Gel, Al, and EDC are the concentrations of gelatin, alginate, and EDC, respectively, all in mg/mL, and (p:a) is the volumetric polymer-to-air foaming ratio.

### 2.2. Compression

A stress-strain curve was obtained for each formulation by compressing the hydrogel samples at a constant rate of 5 mm/min. The test ended when the sample was compressed to 90% strain. The compression modulus was calculated from the linear portion of the stress-strain curve between 10 and 30% strain. None of the samples failed during testing.

Compression was also tested after water uptake. Samples were immersed in DDW for 24 h at 37 °C in an incubator. Since the hydrogel swells in water, swollen sample dimensions were measured using a caliper, and a compression test was performed as described above. At least six repetitions were performed for each formulation.

### 2.3. Water Uptake

Cylindrical samples (12 mm diameter, 4 mm height) were prepared by casting the solutions via the double syringe into silicone molds. One hour after application, the samples were weighed ($w_{i})$ using an analytical balance (Radwag model no. AS 60/220.R2, Crewe, UK) and then submerged in DDW, covered, and placed in a 37 °C incubator. Twenty-four hours later, the samples were taken out of the incubator, blotted dry, and weighed again ($w_{t})$. The water uptake was defined as the weight gained during incubation in water, according to the equation:

(1) $$Water\;uptake\left(\%\right)=\frac{w_{t}-w_{i}}{w_{i}}\times 100$$

At least three repetitions were performed for each formulation.

### 2.4. Resilience

The resilience of the hydrogels was tested by subjecting the samples to 50 cycles of compression to 40% strain and release to the original height at a rate of 5 mm/min. Resilience was measured using the following formula:

(2) $$Resilience\left(\%\right)=\frac{L-U}{L}\times 100$$

where L is the area under the loading stress-strain curve, which indicates the energy required for deformation, and U is the area under the unloading stress-strain curve, which indicates the release of stored energy. L-U is the hysteresis loop, indicating energy dissipation. At least five repetitions were performed for each formulation.

### 2.5. Gelation Time

Gelation time indicates the working time available before the liquid solutions crosslink to become a hydrogel; 1 mL of hydrogel was injected into a 24-well plate with a freely spinning magnet in it. The time required for the magnetic stirrer to stop spinning after injection was defined as the gelation time. At least five repetitions were performed for each formulation.

### 2.6. Cell Viability Evaluation

Cell viability was tested indirectly by exposing human neonatal fibroblasts (Rambam Medical Center, Haifa, Israel) to scaffold extracts, according to the ISO 10993 standard [20] (parts 5 and 12) for the biological evaluation of medical devices. Extracts were prepared by the immersion of samples in cell culture medium for 24 h. The medium was based on Modified Eagle’s Medium (MEM, Sartorius, purchased from Biological Industries, Beit Ha’emek, Israel) supplemented with 10% fetal bovine serum, 1% L-glutamine, and 1% penicillin-streptomycin-nystatin (all purchased from Sigma Aldrich, Rehovot, Israel). Meanwhile, cells were seeded into 96-well plates at a concentration of 5000 cells per well with 0.2 mL cell culture medium. After 24 h of incubation in a humidified 37 °C, 5% CO_2_ environment, the culture medium in the 96-well plates was replaced with the hydrogel extract. The cells were then incubated in the extracts for an additional 24 or 48 h. The AlamarBlue™ assay (Invitrogen™, Rhenium, Modi’in, Israel) was used to quantify the number of cells that remain viable after incubation in the extracts, using a spectrophotometer (Spectra Max 340 PC384, Molecular Devices, San Jose, CA, USA). Cells cultured in fresh medium served as the control.

### 2.7. Statistical Analysis

All data were processed using Microsoft Excel. Statistical analysis was performed using ANOVA (Tukey Kramer post hoc) via IBM SPSS (v. 27). A *p* value of <0.05 and a *p* value of <0.001 is marked with an * and ** in the figures, respectively.

## 3. Results and Discussion

The effects of the formulation parameters (polymer concentration, foaming ratio, and crosslinking degree) on the hydrogel’s properties were studied. Cellular viability was also tested on mouse preadipocytes and human fibroblasts. The mechanical, physical, and biological characterization of the hydrogel provides a broad picture of which tissues these formulations can act as a scaffold for. It is possible to achieve a wide range of properties, as can be seen in the following results.

### 3.1. Compression

**Figure 2:**
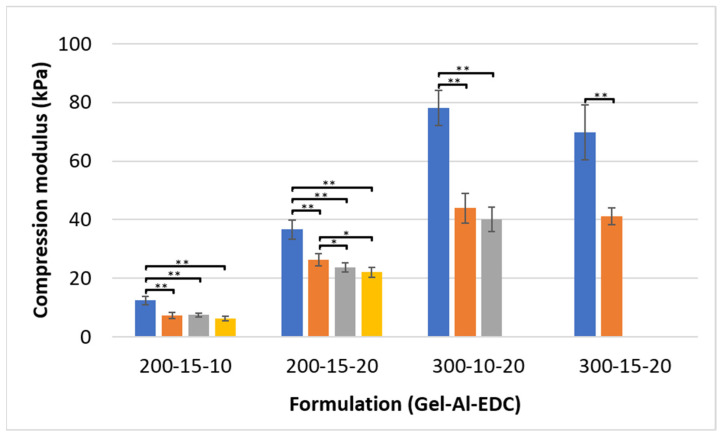
Compression modulus of hydrogel cylinders with various concentrations of gelatin, alginate, and EDC (Gel-Al-EDC). Foaming ratios (polymer:air) are represented by different bar colors: ■ non-foamed; ■ 2:1; ■ 1.5:1; ■ 1:1. A *p* value of <0.05 and <0.001 is marked with an * and **, respectively.

The compression modulus of the various hydrogels is presented in Figure 2. A wide range of stiffnesses can be achieved by varying the gelatin, alginate, and EDC concentrations as well as the foaming ratios. Non-foamed hydrogels with relatively low gelatin and EDC concentrations (200 mg/mL and 10 mg/mL, respectively) exhibited a compression modulus of 12.4 kPa. As gelatin and EDC concentrations increased to 300 mg/mL and 20 mg/mL, respectively, the modulus reached a higher value of 78.2 kPa. These values decreased as air was added into the formulations via the foaming ratios, although the difference in compression modulus between formulations with different foaming ratios was minor.

The observed decrease in modulus upon the addition of air to hydrogels can be attributed to the reduced polymer content in the final scaffold. When air is added to a uniformly shaped sample, it effectively decreases the polymer content in the sample. The air pockets do not resist mechanical loads, and, additionally, they provide spaces into which the polymeric walls can compress. These factors collectively contribute to the lower compression moduli observed in the foamed hydrogels.

Figure 2 also shows that increasing the concentration of the crosslinker from 10 to 20 mg/mL and increasing the gelatin content from 200 to 300 mg/mL increases the modulus, while the increase in alginate concentration slightly decreases it. The subtle change attributed to the alginate probably results from the low concentrations of alginate used. Therefore, a higher relative alginate concentration may result in a more pronounced effect.

Song et al. have also shown that a less crosslinked network, along with an increase in the porosity of crosslinked collagen scaffolds, leads to a lower compression modulus [21]. They reported a compression modulus of approximately 5.8 MPa in the less porous and more crosslinked scaffold and 0.9 MPa in the more porous and less crosslinked scaffold. Matching the mechanical properties of the scaffold with the mechanical properties of the target tissue is crucial for successful mechanostimulation, the initiation of relevant signal pathways, and, ultimately, cell differentiation into the required cell type [1,6,7]. Both values are much higher than the moduli reported here, even though the hydrogels are from the same family, making them unsuitable for soft tissue applications.

A compression modulus as low as 6–7 kPa can be achieved by the 20015–10 foamed formulations. These formulations are particularly suitable for very soft tissues, such as lung tissue [22], trachea and large bronchi [23], or within the oral cavity, the soft palate, and tongue [24,25,26]. Foamed formulations containing 300 mg/mL gelatin reach a much higher modulus within the 40–44 kPa range. These formulations exhibit mechanical properties more comparable to glandular breast tissue [27,28]. While non-foamed formulations can achieve even higher moduli, they may not be as effective for tissue ingrowth due to the absence of the pores required for cellular infiltration and adhesion. It is important to note that foamed samples based on 300 mg/mL gelatin exhibit 80% water uptake and, as a result, their modulus decreases to approximately 20 kPa, which makes them suitable for cardiac applications [29,30].

A compression modulus of 29.9 kPa was recently reported in dopamine-grafted hyaluronic acid and methacrylated hyaluronic acid dual network injectable hydrogels, but resilience was not tested for this partly synthetic system [31]. Another recent research describes electroconductive gelatin-alginate hydrogels for tissue engineering [32]. Although the base materials are similar, the compression modulus of the hydrogel is in the range of 400 kPa and increases further with the addition of the carbon nanofibers for conductivity purposes.

### 3.2. Water Uptake

**Figure 3:**
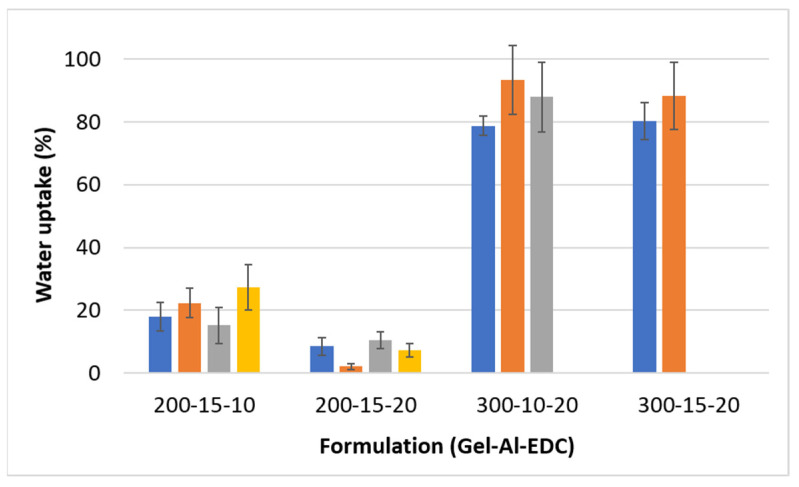
Water uptake as percentage weight gain in 24 h as a measure of water uptake of hydrogels with different concentrations of gelatin, alginate, and EDC (Gel-Al-EDC). Foaming ratios (polymer:air) are represented by different colors: ■ non-foamed; ■ 2:1; ■ 1.5:1; ■ 1:1.

The polymers used in this study are hydrophilic. Therefore, when the hydrogels are injected in vivo, they absorb liquid from the environment, leading to swelling. Swelling affects both the dimensions and the mechanical properties of the hydrogel. Water uptake is measured by the percentage weight gain and serves as an indicator of the hydrogel’s swelling degree (Figure 3).

Within each formulation, there are no significant differences between the foaming ratios. This lack of variation was expected because any excess water that may accumulate in the pores is removed from the sample prior to weighing. Consequently, the weight change is only determined by the swelling of the polymer itself. Foaming by itself does not change the polymer’s characteristics and therefore the swelling degree remains the same. Additionally, the water penetration into the bulk of the hydrogel after 24 h is not affected by the presence of pores due to the high hydrophilicity of gelatin and alginate.

Different formulations result in different water uptake levels. Hydrogels containing 200 mg/mL gelatin swell significantly less than those containing 300 mg/mL gelatin due to the higher hydrophilic nature of the samples. Also, a comparison of the 10 mg/mL EDC and 20 mg/mL EDC within the 200–15 Gel-Al formulation shows that a higher crosslinker concentration results in reduced swelling. This is likely due to the higher crosslinking density. Mechanical tests also show that a higher crosslinker concentration increases the modulus (Figure 2). The combination of the two results confirms that the crosslinking agent is the limiting factor in the reaction. Therefore, the addition of a higher concentration of EDC results in a higher crosslinking density and a denser network, which is less available for water absorption.

As the scaffold is situated in an aqueous environment, some weight loss is expected as the hydrogel degrades over time. Previous research conducted by our group demonstrated that non-porous 400–10–15 mg/mL gel-al-EDC exhibited approximately 9% weight loss after 24 h, resulting mainly from the diffusion of small molecules as water enters the sample [33].

### 3.3. Compression of Swollen Samples

**Figure 4:**
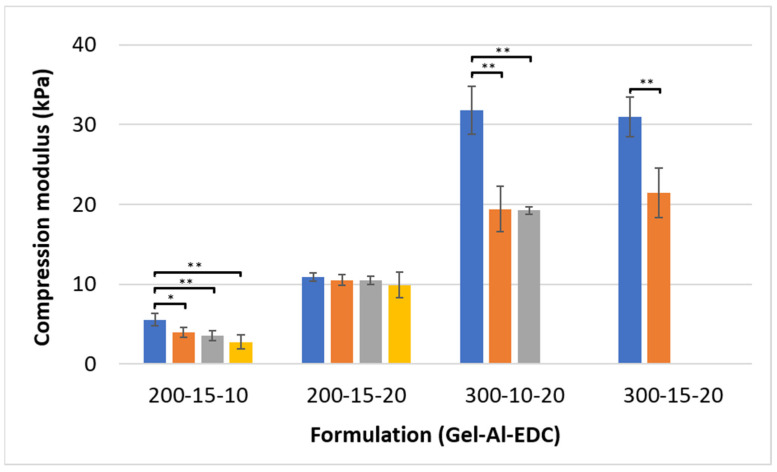
Compression modulus of hydrogel cylinders after 24 h of immersion in water. Hydrogel formulations are noted on the *x*-axis as Gel-Al-EDC, and foaming ratios (polymer:air) are represented by different bar colors: ■ non-foamed; ■ 2:1; ■ 1.5:1; ■ 1:1. A *p* value of <0.05 and <0.001 is marked with an * and **, respectively.

Figure 4 presents the compression modulus of hydrogel samples after swelling in an aqueous environment. The compression modulus of non-foamed samples varies from 5.5 kPa for the 200–15–10 formulation to 31.8 kPa for the 300–10–20 formulation. It reaches a value as low as 2.7 kPa for the 200–15–10 (1:1) foamed samples. As was observed also in un-swollen samples (Figure 2), the addition of air generally reduces the compression modulus in most formulations, but the foaming ratios only slightly affected this trend.

Swelling reduces the compression modulus. Characterizing the mechanical properties of swollen samples allows us to gain an understanding of material properties in vivo, where the environment is aqueous. After water uptake, the low gelatin and low EDC formulations reach notably low values. These hydrogels can be compatible for use in white and gray matter in the brain, where the native tissue has a modulus of between 2.8 and 3.6 kPa [34], or adipose tissue in the breast with a modulus of between 2 and 4 kPa [35,36].

**Figure 5:**
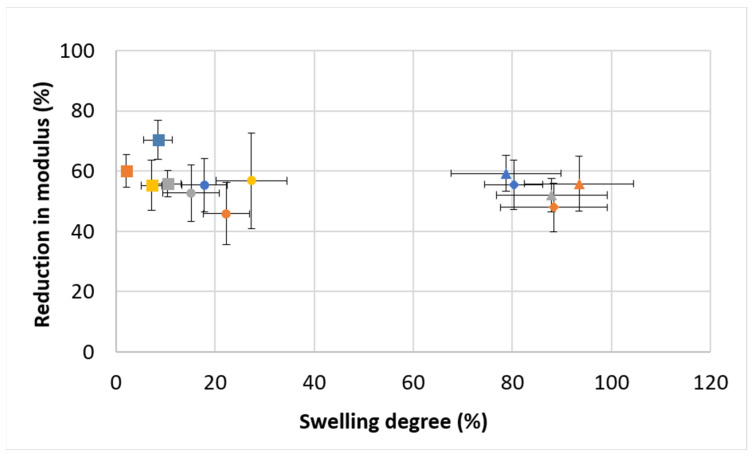
Reduction in compression modulus (%) as affected by the swelling degree. Formulations (Gel-Al-EDC) are represented by different bullet shapes: ● 200–15–10; ■ 200–15–20; ▲300–10–20; ♦ 300–15–20. Foaming ratios (polymer:air) are represented by different colors: blue for non-foamed; orange for 2:1; gray for 1.5:1; yellow for 1:1.

Figure 5 shows the effect of the swelling degree on the decrease in compression modulus (%). It is interesting to see that regardless of the swelling degree, which can be low (approximately 10%) or high (approximately 90%), the reduction in modulus is always in the range of 45–60%. This may be attributed to the homogeneity of the polymeric network. In a homogeneously crosslinked network, the distribution of polymer chains and crosslinks is even. As water is absorbed into the hydrogel during swelling, it distributes evenly across the polymer network. As the polymer network expands uniformly, it leads to a similar disruption in chain interactions, resulting in a consistent reduction in modulus. The mechanical properties of hydrogels are strongly related to polymer chain interactions, specifically friction. The addition of water creates larger distances between adjacent chains, thus lowering the friction and stiffness [37].

### 3.4. Resilience

Many tissues in the body undergo repetitive motion. For example, lungs expand and contract with every breath, and adipose tissue all over the body constantly experiences compression and release. It is therefore critical that the scaffold not only endures this motion without failure but also does not undergo significant plastic deformations.

**Figure 6:**
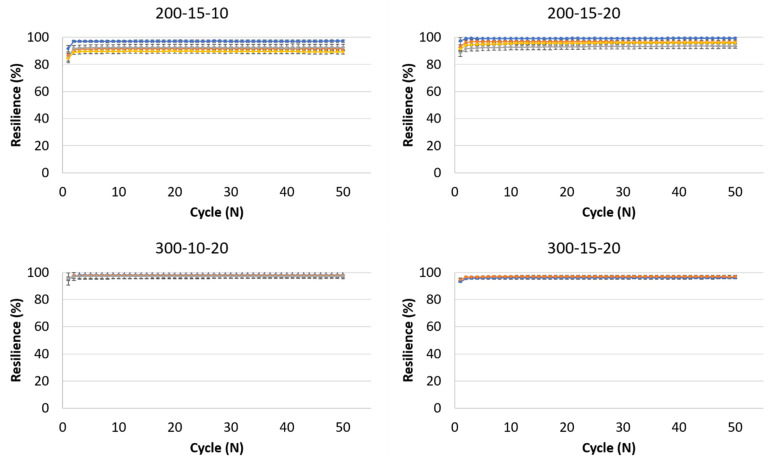
Resilience of the hydrogels during 50 loading and unloading cycles. The formulations are noted above each graph, and foaming ratios (polymer:air) are represented by different colors: ■ non-foamed; ■ 2:1; ■ 1.5:1; ■ 1:1.

Figure 6 shows the resilience of the hydrogels studied in our research. All tested formulations exhibited remarkably high resilience. After a slight preconditioning, which is evident in the first cycles [38,39], there is nearly no energy dissipation throughout the cycles. Resilience is as high as 99% in some of the non-foamed formulations. It was previously mentioned that the porosity of the hydrogels plays a crucial role in tissue regeneration scaffolds, and even the porous scaffolds reached a resilience higher than 96% (a 2:1 foaming ratio in all formulations containing 20 mg/mL EDC). The slightly lower resilience of the foamed samples, compared to the high resilience of the non-foamed samples, probably results from their overall lower polymer content. Decreasing the polymeric content and replacing it with air within the pores decreases the resilience as well. It is important to note that even after this slight decrease, resilience is still very high. Other natural polymers, such as elastin and resilin, have a resilience of 90 and 92%, respectively [40,41]. These are considered highly resilient, meaning that our gelatin-based hydrogel is highly resilient as well.

Decreasing the EDC concentration also results in lower resilience, as observed for the 200–15–10 hydrogel. The lower EDC concentration results in a less crosslinked network, leading to a sparser polymeric network. Consequently, this means that there is a relatively more uncrosslinked polymer that does not retain its shape during loading.

Recent research by Han et al. reported a xylan-loaded polyvinyl alcohol and sodium trimetaphosphate-based hydrogel that can maintain up to 82% of the original stress after 1000 compression cycles [42]. Another recent paper by Han et al. reported resilience of 90–94% in their acrylic acid and tri(ethylene glycol) diacrylate-dopamine hydrogel [11]. The hydrogels reported here show resilience either in the same range or higher, while having a lower compression modulus, representing their possible use in softer tissues. It is important to note that most research regarding hydrogel resilience has reported testing only a few cycles [11,43,44,45], while the scaffolds reported here maintain high resilience for at least 50 repetitions, indicating that they are suitable for use in repetitive motion. Testing an even higher number of cycles would confirm their mechanical compatibility use in applications such as cardiac and lung tissue regeneration.

### 3.5. Gelation Time

**Figure 7:**
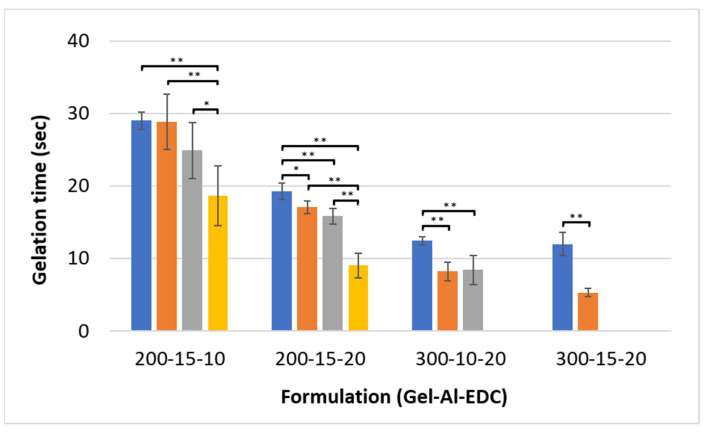
Gelation time of the hydrogels. Different formulations are noted on the *x*-axis as Gel-Al-EDC. Foaming ratios (polymer:air) are represented by different colors: ■ non-foamed; ■ 2:1; ■ 1.5:1; ■ 1:1. A *p* value of <0.05 and <0.001 is marked with an * and **, respectively.

As shown in Figure 7, the gelation time of all studied formulations is in the range of 5–30 s. Increasing both gelatin and crosslinker concentrations leads to faster gelation times. The gelation time of hydrogels is influenced by two key factors: crosslinking kinetics and solution viscosity. Increasing the crosslinker concentration (e.g., from 10 mg/mL to 20 mg/mL in the 200–15–10 and 200–15–20 hydrogels) accelerates the crosslinking reaction. There are more EDC molecules acting simultaneously, leading to a rapid linking of polymer chains that translates to a quicker transition from solution to gel. This also confirms the crosslinker as the limiting factor, as observed in Figure 2. Additionally, as more air is incorporated into the hydrogel (smaller p:a value), the gelation time decreases.

From a practical standpoint, during the measurement, as long as the magnet spins, the hydrogel is considered injectable. Hence, the method used in this study for evaluating the gelation time mimics real-world clinical use, where the hydrogel is injected and has to be manipulated to fill the void’s irregular shape. As the crosslinking reaction rate increases, there is less time for the hydrogel to completely fill the targeted area, whereas very low solution viscosity can cause the polymer to flow away from the desired location. Therefore, optimizing the combination of viscosity and crosslinker concentration becomes crucial for achieving a gelation time that is acceptable to the clinician.

Gelation time requirements vary between different applications. For example, hydrogels that are used as hemostats or for treating perforation, such as in the gastrointestinal tract, should have an ultra-fast gelation time of approximately 5 s, so that they successfully form a seal and prevent the leakage of contents [46,47]. In contrast, hydrogel scaffolds for wound healing applications, such as skin lacerations, require a significantly slower gelation time, closer to 30 s for better surface adhesion and easier application [48]. Matching the appropriate gelation time with the desired mechanical properties for the target tissue plays a major role in developing a suitable in situ crosslinking hydrogel.

**Figure 8:**
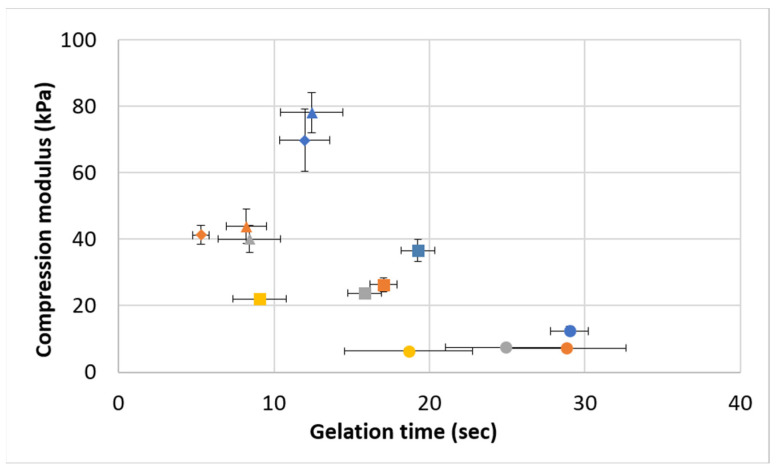
The relationship between the compression modulus and gelation time. Formulations (Gel-Al-EDC) are represented by different bullet shapes: ● 200–15–10; ■ 200–15–20; ▲300–10–20; ♦ 300–15–20. Foaming ratios (polymer:air) are represented by different colors: blue for non-foamed; orange for 2:1; gray for 1.5:1; yellow for 1:1.

Figure 8 shows the relationship between gelation time and the compression modulus. It is notable that a variety of hydrogels, in terms of gelation times and stiffness (modulus), were obtained using the studied formulations. These include combinations of a high modulus (70–80 kPa) with a short gelation time (12–13 s), a medium modulus (40–44 kPa) with a very short gelation time (5–8 s), a medium modulus (20–27, 34 kPa) with a short or medium gelation time (8–19 s), and a low modulus (7–12 kPa) with a medium or long gelation time (17–28 s). The only hydrogel types that do not exist in the current study are those that combine a high modulus with a long gelation time and a low modulus with a short gelation time.

The general trend is that formulations that yielded stiffer hydrogels exhibited shorter gelation times. The two factors that decrease the gelation time are the initial polymeric solution’s viscosity, governed by gelatin and alginate concentrations, and the rate of crosslinking, governed by the EDC concentration and the polymer-to-air ratio. When both are increased, a stiffer hydrogel is also formed. This explains why we did not achieve extreme combinations of a high modulus with a long gelation time and a low modulus with a short gelation time. It should be emphasized that the broad range of moduli obtained for the studied formulations actually covers the broad range of applications mentioned above (Figure 1), and a suitable gelation time can be fitted for most of them.

### 3.6. Formulation-Properties Effects

**Figure 9:**
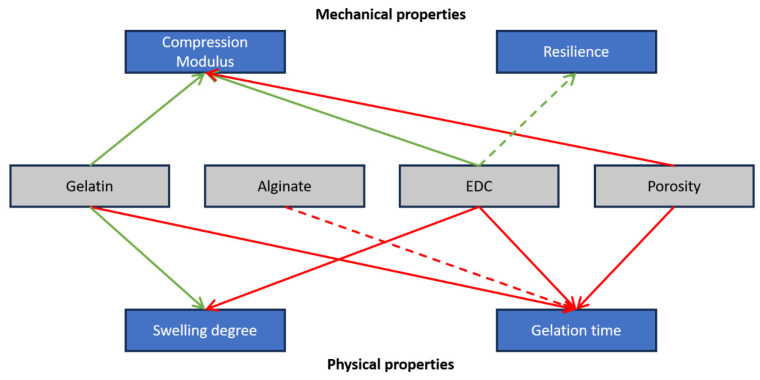
Qualitative model illustrating the relationships between hydrogel components and its properties. Red arrows indicate a decreasing trend, and green arrows indicate an increasing trend. A solid line indicates a strong effect, and a dashed line indicates a weak effect.

A qualitative model illustrating the interconnected effects of the formulation parameters on the mechanical properties (modulus and resilience) and on the physical properties (gelation time and swelling degree) is summarized in Figure 9, with green arrows indicating an increase trend and red arrows indicating a decrease trend.

The most dominant formulation parameters are the gelatin and EDC concentrations. An increase in both results in higher modulus and lower gelation time, i.e., a stiffer hydrogel that is created in a shorter time. The water uptake is increased when using a higher gelatin concentration and is decreased when using a higher EDC concentration due to the creation of a denser crosslinked network. A higher EDC concentration also slightly increases resilience for the same reason.

The polymer-to-air ratio also affects the hydrogel’s properties. The main trends obtained are lower modulus and gelation time, obtained due to the lower polymer-to-air ratio, i.e., higher porosity. Alginate is added in small amounts, and therefore it only slightly affects the gelation time, and thus enables its fine tuning.

This model provides a general view of the relationships between the formulation parameters and the most important hydrogel’s properties, emphasizing the ability to control the behavior of the hydrogel for specific tissue engineering applications.

### 3.7. Biological Characterization

The polymers used in this study are biocompatible and have little to no environmental toxicity, as they themselves are used for contaminant removal [49,50]. EDC is also considered to be a more biocompatible crosslinker than the commonly used glutaraldehyde and formaldehyde [51]. Tissue adhesives for wound healing, based on 400 mg/mL gelatin, 10 mg/mL alginate, and 20 mg/mL EDC, were tested in a porcine model, and no adverse effects were observed within 14 days [52]. Therefore, a cytotoxic effect was not expected in this study as well.

**Figure 10:**
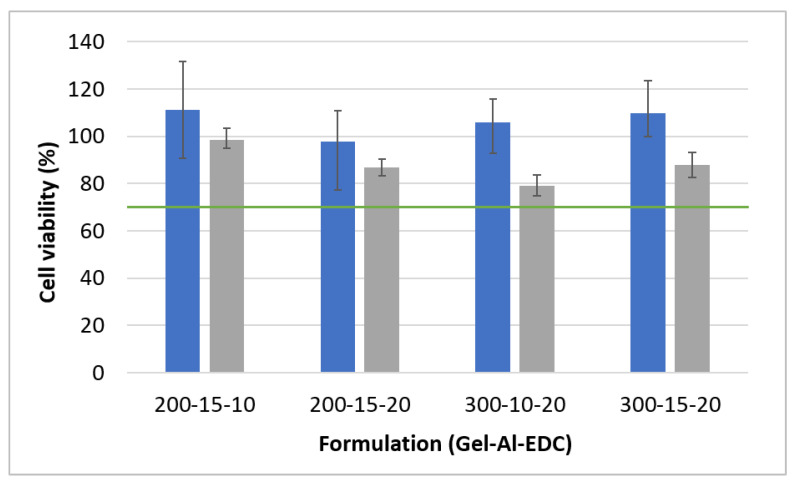
Cell viability of non-foamed hydrogels on human fibroblasts. Blue and gray bars show the results after 24- and 48-h incubation of the cells in hydrogel extracts, respectively. The green line marks 70% viability, which is considered by the FDA as the limit of cytotoxicity.

To assess potential cytotoxicity, indirect cytotoxicity tests were performed on human neonatal fibroblasts using an AlamarBlue™ assay. The results are presented in Figure 10. The US Food and Drug Administration (FDA) guidelines based on ISO 10993 classify hydrogels that result in a decrease in cell viability greater than 30% as cytotoxic. All tested hydrogels maintained over 70% cell viability, indicating no cytotoxicity. According to ISO 10993, using a foamed hydrogel would result in using less weight per ml of extract medium; therefore, only non-foamed hydrogels with the highest density were tested to consider the ‘worst-case scenario’. Since foamed hydrogels contain less polymer and crosslinker, we expect them to be even more biocompatible.

### 3.8. Future Research

Tissue regeneration is a long-term process and the rate of tissue regeneration differs between tissues. This must be taken into consideration when designing a scaffold for tissue regeneration. In vivo experiments using similar formulations as tissue adhesives for the closure of porcine skin incisions showed rapid healing and a higher degree of wound closure compared to the control groups (sutured incisions and Hystoacryl^®^, (B. Braun Surgical, S.A., Barcelona, Spain)). These results suggest a degradation rate that is well-suited for tissue healing, neither too slow nor too fast [52]. The hydrogel degradation rate is expected to vary between different formulations due to the different gelatin/crosslinker ratios, and it must match the rate of tissue regeneration. Therefore, it needs to be tested in vitro and in vivo.

Another direction for future research relates to the high water uptake found in formulations containing 300 mg/mL gelatin. A water uptake of approximately 80% changes the dimensions of the hydrogel, and if the scaffold is used in a confined space, it may apply forces to the surrounding tissues, possibly altering the natural environment. This, along with cellular differentiation tests, should also be conducted to ensure that even though the mechanical properties match the target tissue, it is possible to promote the specific cell signaling pathway necessary for the regeneration of a specific tissue in vivo.

## 4. Conclusions

Our research introduces a novel, highly resilient, biodegradable, and customizable injectable scaffold for soft tissue regeneration. Leveraging the advantages of natural polymers, we address a critical challenge in scaffold design: achieving a range of stiffnesses while maintaining high scaffold resilience. Our results present porous hydrogels with a compressive modulus ranging from a low 2.7 kPa to over 40 kPa. These hydrogels exhibit extraordinary resilience, exceeding 90% for all studied formulations.

We reported a tailorable platform, which enabled us to achieve properties fitting for use in various soft tissues. The studied hydrogels demonstrated high biocompatibility when tested on human fibroblasts, confirming their suitability for in vivo applications. This study focused mainly on the mechanical and physical properties and confirmed a lack of cytotoxicity. Future research will include direct cell viability tests as well as long-term cellular compatibility and differentiation evaluations of various types of cells on the scaffolds. Animal studies will be performed as well.

When used as a scaffold for tissue regeneration applications, the most important hydrogel mechanical properties are its compression modulus and resilience. The former, which is a measure of the stiffness, should be similar to that of the surrounding tissue to enable proper functioning. The latter should be very high to withstand a large number of cyclic loads without significant plastic deformation. Our results indicate that the modulus is affected mainly by the gelatin and EDC concentrations and also by the polymer-to-air ratio.

The most important hydrogel physical properties are the gelation time, which should fit proper manipulation by the physician during the surgery, and the water uptake, which affects both the dimensions and the mechanical properties of the swollen hydrogel. These two physical properties are affected by both gelatin and EDC concentration, and the gelation time is affected also by the porosity (polymer-to-air ratio) of the hydrogel. A broad range of gelation times (5–30 s) were obtained.

In summary, our natural polymer-based hydrogel scaffolds offer a promising and versatile solution for soft tissue regeneration applications, combining high performance and biocompatibility to address a wide range of clinical needs. A thorough understanding of the formulation-properties effects, which was achieved in the current study, will enable us to customize these hydrogel scaffolds for various specific applications.

## Figures and Tables

**Table 1:** The hydrogel formulations used in the current study. Foaming ratios are presented as volumetric polymer-to-air ratios (p:a).

Gelatin (mg/mL)	Alginate (mg/mL)	EDC (mg/mL)	Foaming Ratio (p:a)
200	15	10, 20	Non-foamed, 2:1, 1.5:1, 1:1
300	10	20	Non-foamed, 2:1, 1.5:1
300	15	20	Non-foamed, 2:1

## Data Availability

The original contributions presented in the study are included in the article, further inquiries can be directed to the corresponding author.
